# Crystal structure of (*E*)-2,6-dimeth­oxy-4-{[(4-meth­oxy­phen­yl)imino]­meth­yl}phenol

**DOI:** 10.1107/S2056989018013713

**Published:** 2018-10-05

**Authors:** Md. Serajul Haque Faizi, Mohamad Nadeem Lone, Necmi Dege, Sergey Malinkin, Tatiana Yu. Sliva

**Affiliations:** a Department of Chemistry, Langat Singh College, Babasaheb Bhimrao Ambedkar Bihar University, Muzaffarpur, Bihar, India; b Department of Chemistry, Govt. College For Women, Udhampur, Jammu and Kashmir 182 101, India; c Ondokuz Mayıs University, Arts and Sciences Faculty, Department of Physics, Atakum 55139 Samsun, Turkey; d Department of Chemistry, National Taras Shevchenko University of Kiev, 64/13, Volodymyrska Street, City of Kyiv, 01601, Ukraine

**Keywords:** crystal structure, syringaldehyde, 4-meth­oxy­aniline, 4-hy­droxy-3,5-di­meth­oxy­benzaldehyde

## Abstract

In the title compound, C_16_H_17_NO_4_, the dihedral angle between benzene rings is 72.7 (2)°. The meth­oxy groups are rotated by 2.4 (2) and −4.9 (2) (benzil­idene moiety) and by 5.6 (3)° (aniline moiety) relative to the adjacent benzene ring. In the crystal, the mol­ecules are linked into chains along [101] through C—H⋯O and O—H⋯N hydrogen bonds.

## Abstract 2

The title mol­ecule is comprised of two non-coplanar benzene rings connected by an imino group in a *trans*-configuration. In the crystal, the mol­ecules are linked *via* O—H⋯N and C—H⋯O hydrogen bonds, forming chains along [101].

## Chemical context   

Syringaldehyde is a product of the catalytic decomposition of lignin (Crestini *et al.*, 2010 ▸). Syringaldehyde is widely used as a mol­ecular marker to monitor pollution sources and detect the extent of combustion (Robinson *et al.*, 2006 ▸). It is also known to be an anti­oxidant (Ibrahim *et al.*, 2012 ▸), anti­cancer, anti-inflammatory (Duke, 2003 ▸) and anti­fungal agent (Gurpilhares *et al.*, 2006 ▸). In addition, its Schiff bases are known to exhibit a wide range of biological activities (Shi & Zhou, 2011 ▸; da Silva *et al.*, 2011 ▸).
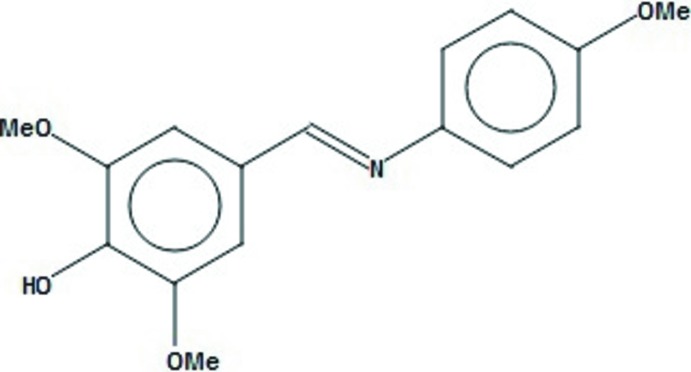


## Structural commentary   

**Figure 1:**
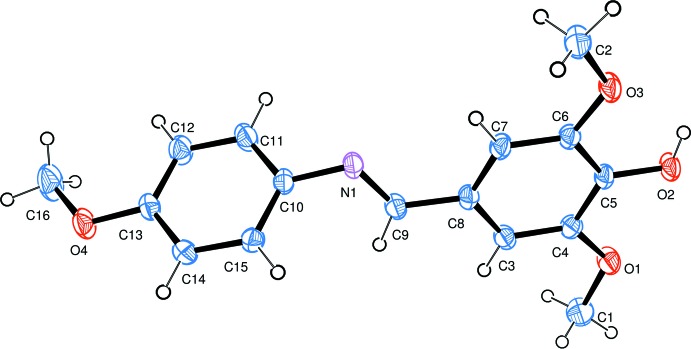
A view of the mol­ecular structure of the title compound, with the atom labelling. Displacement ellipsoids are drawn at the 40% probability level.

The mol­ecular structure of the title molecule is shown on Fig. 1 ▸. The compound has a *trans*-configuration of the C9=N1 double bond. The mol­ecule has a non-planar conformation with the two benzene rings forming a dihedral angle of 72.7 (2)°. The meth­oxy groups are almost co-planar with the planes of the adjacent aromatic rings [the C1—O1—C4—C3, C2—O3—C6—C7 and C16—O4—C13—C12 torsion angles are −4.9 (2), 2.4 (2) and 5.6 (3)°, respectively].

## Supra­molecular features   

**Figure 2:**
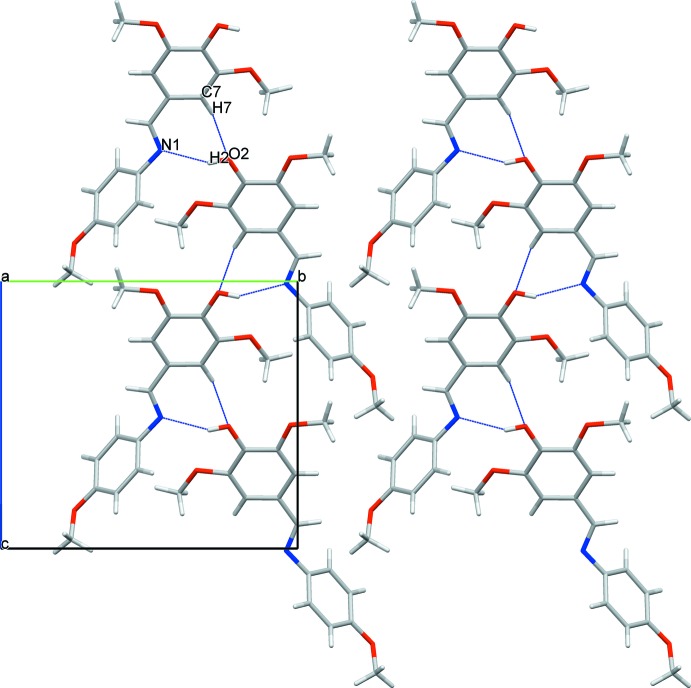
A view along the *a* axis of the crystal packing. Dashed lines indicate hydrogen bonds (see Table 1 ▸).

In the crystal, the mol­ecules are connected *via* C7—H7⋯O2^ii^ and O2—H2⋯N1^i^ hydrogen bonding (Table 1 ▸), forming chains along the [101] direction (Fig. 2 ▸).

## Database survey   

A search of the Cambridge Structural Database (CSD version 5.39, update of May 2018; Groom *et al.*, 2016 ▸) revealed the structures of five similar Schiff bases based on *p*-meth­oxy­aniline and *p*-hy­droxy­benzaldehyde: 4-[(4-meth­oxy­phenyl­imino)­meth­yl]phenol, (I) (VUKDEK; Yeap *et al.*, 1992 ▸), (*E*)-5-meth­oxy-2-[(4-meth­oxy­phenyl­imino)­meth­yl]phenol, (II) (NURNAQ; Sahin *et al.*, 2010 ▸), 2-meth­oxy-4-{[(4-meth­oxy­phen­yl)imino]­meth­yl}phenol, (III) (MOTLIR; Singh *et al.*, 2008 ▸), 2,6-di-*tert*-butyl-4-[(4-meth­oxy­phenyl­imino)­meth­yl]phenol, (IV) (WEFTEH; Xin *et al.*, 2006 ▸) and 5-bromo-2-meth­oxy-4-{[(4-meth­oxy­phen­yl)imino]­meth­yl}phenol monohydrate, (V) (GAPFEK; Mao *et al.*, 2012 ▸). The dihedral angle between the benzene rings in the title compound [72.7 (2)°] is larger than those in compounds (I), (III) and (IV) (49.75–53.63°). Compounds (II) and (V) are almost planar. In all of the compounds, the meth­oxy groups deviate from the plane of aromatic system. There are no C—H⋯π or π–π inter­actions in the crystal structure of the title compound, in contrast to what is observed for compounds (I), (IV) and (V).

## Synthesis   

4-Hy­droxy-3,5-di­meth­oxy­benzaldehyde (syringaldehyde) (0.05 mol) was added to a mixture of 50 ml of methanol and *p*-meth­oxy­aniline (PMA) (5 ml, 0.05 mol) and 50 ml of distilled water. The reaction mixture was taken in a clean 250 ml round-bottom flask and stirred well with a magnetic stirrer. It was then refluxed for 7 h. The dark-yellow product that formed was separated by filtration, dried under vacuum and recrystallized from methanol solution upon slow evaporation for two days (yield 65%, m.p. 353–357 K).

## Refinement   

Crystal data, data collection and structure refinement details are summarized in Table 2 ▸. H atoms were positioned geom­etrically and refined using a riding model: O—H = 0.82–0.96 Å and C—H = 0.93–0.96 Å with *U*_iso_(H) = 1.2*U*_eq_(C) or 1.5*U*_eq_(O, Cmethyl).

## Supplementary Material

Crystal structure: contains datablock(s) I. DOI: 10.1107/S2056989018013713/ld2146sup1.cif

Supporting information file. DOI: 10.1107/S2056989018013713/ld2146Isup3.cml

Structure factors: contains datablock(s) I. DOI: 10.1107/S2056989018013713/ld2146Isup3.hkl

CCDC reference: 1843910

Additional supporting information:  crystallographic information; 3D view; checkCIF report

## Figures and Tables

**Table 1:** Hydrogen-bond geometry (Å, °)

*D*—H⋯*A*	*D*—H	H⋯*A*	*D*⋯*A*	*D*—H⋯*A*
O2—H2⋯N1^i^	0.82	2.21	2.9415 (18)	149
C7—H7⋯O2^ii^	0.93	2.29	3.2043 (18)	167

Symmetry codes: (i) 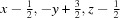; (ii) 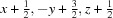.

**Table 2:** Experimental details

Crystal data	
Chemical formula	C_16_H_17_NO_4_
*M*_r_	287.30
Crystal system, space group	Monoclinic, *P*2_1_/*n*
Temperature (K)	296
*a*, *b*, *c* (Å)	10.4996 (15), 12.4896 (18), 11.8128 (17)
β (°)	107.936 (5)
*V* (Å^3^)	1473.8 (4)
*Z*	4
Radiation type	Mo *K*α
μ (mm^−1^)	0.09
Crystal size (mm)	0.45 × 0.33 × 0.21

Data collection	
Diffractometer	Bruker APEXII CCD
No. of measured, independent and observed [*I* > 2σ(*I*)] reflections	19289, 2887, 2306
*R*_int_	0.035
(sin θ/λ)_max_ (Å^−1^)	0.617

Refinement	
*R*[*F* ^2^ > 2σ(*F* ^2^)], *wR*(*F* ^2^), *S*	0.044, 0.116, 1.05
No. of reflections	2887
No. of parameters	194
H-atom treatment	H-atom parameters constrained
Δρ_max_, Δρ_min_ (e Å^−3^)	0.17, −0.21

Computer programs: *APEX2* and *SAINT* (Bruker, 2004 ▸), *SHELXS97* (Sheldrick 2008 ▸), *SHELXL2017* (Sheldrick, 2015 ▸), *ORTEP-3 for Windows* (Farrugia, 2012 ▸), *Mercury* (Macrae *et al.*, 2008 ▸) and *PLATON* (Spek, 2009 ▸).

## supplementary crystallographic information

### Crystal data

**Table:**

C_16_H_17_NO_4_	*F*(000) = 608
*M**_r_* = 287.30	*D*_x_ = 1.295 Mg m^−^^3^
Monoclinic, *P*2_1_/*n*	Mo *K*α radiation, λ = 0.71073 Å
*a* = 10.4996 (15) Å	Cell parameters from 6353 reflections
*b* = 12.4896 (18) Å	θ = 2.3–28.3°
*c* = 11.8128 (17) Å	µ = 0.09 mm^−^^1^
β = 107.936 (5)°	*T* = 296 K
*V* = 1473.8 (4) Å^3^	Prism, colorless
*Z* = 4	0.45 × 0.33 × 0.21 mm

### Data collection

**Table:**

Bruker APEXII CCD diffractometer	*R*_int_ = 0.035
φ and ω scans	θ_max_ = 26.0°, θ_min_ = 2.3°
19289 measured reflections	*h* = −12→12
2887 independent reflections	*k* = −15→15
2306 reflections with *I* > 2σ(*I*)	*l* = −14→14

### Refinement

**Table:**

Refinement on *F*^2^	0 restraints
Least-squares matrix: full	Hydrogen site location: inferred from neighbouring sites
*R*[*F*^2^ > 2σ(*F*^2^)] = 0.044	H-atom parameters constrained
*wR*(*F*^2^) = 0.116	*w* = 1/[σ^2^(*F*_o_^2^) + (0.0509*P*)^2^ + 0.4295*P*] where *P* = (*F*_o_^2^ + 2*F*_c_^2^)/3
*S* = 1.05	(Δ/σ)_max_ < 0.001
2887 reflections	Δρ_max_ = 0.17 e Å^−^^3^
194 parameters	Δρ_min_ = −0.20 e Å^−^^3^

### Special details

**Table:**

Geometry. All esds (except the esd in the dihedral angle between two l.s. planes) are estimated using the full covariance matrix. The cell esds are taken into account individually in the estimation of esds in distances, angles and torsion angles; correlations between esds in cell parameters are only used when they are defined by crystal symmetry. An approximate (isotropic) treatment of cell esds is used for estimating esds involving l.s. planes.

### Fractional atomic coordinates and isotropic or equivalent isotropic displacement parameters (Å^2^)

**Table:**

	*x*	*y*	*z*	*U*_iso_*/*U*_eq_	
O2	0.34000 (12)	0.73324 (9)	0.04196 (10)	0.0465 (3)	
H2	0.347012	0.797633	0.056037	0.070*	
O3	0.52685 (12)	0.84064 (9)	0.20703 (10)	0.0473 (3)	
O1	0.32775 (12)	0.52385 (9)	0.03728 (11)	0.0530 (4)	
O4	1.04377 (13)	0.24711 (10)	0.85736 (10)	0.0524 (3)	
N1	0.76944 (13)	0.53851 (10)	0.50837 (11)	0.0388 (3)	
C10	0.83876 (15)	0.46251 (12)	0.59633 (13)	0.0350 (4)	
C15	0.93325 (16)	0.39274 (12)	0.57930 (14)	0.0369 (4)	
H15	0.951974	0.392859	0.507304	0.044*	
C8	0.61237 (15)	0.56465 (13)	0.31098 (13)	0.0355 (4)	
C4	0.42672 (15)	0.56804 (13)	0.12973 (13)	0.0371 (4)	
C6	0.53028 (15)	0.73189 (12)	0.21930 (13)	0.0344 (4)	
C14	1.00014 (16)	0.32282 (12)	0.66827 (14)	0.0384 (4)	
H14	1.064957	0.277361	0.656291	0.046*	
C7	0.61974 (15)	0.67549 (13)	0.30970 (13)	0.0361 (4)	
H7	0.684472	0.711472	0.369303	0.043*	
C9	0.69704 (15)	0.50052 (13)	0.40957 (14)	0.0377 (4)	
H9	0.697878	0.426753	0.399120	0.045*	
C5	0.43155 (15)	0.67913 (13)	0.13033 (13)	0.0345 (4)	
C3	0.51728 (16)	0.51059 (13)	0.21959 (14)	0.0386 (4)	
H3	0.514739	0.436151	0.219040	0.046*	
C13	0.97179 (16)	0.31969 (13)	0.77481 (14)	0.0380 (4)	
C11	0.81296 (18)	0.46051 (15)	0.70406 (15)	0.0486 (5)	
H11	0.751385	0.508458	0.717490	0.058*	
C12	0.87678 (18)	0.38867 (16)	0.79243 (16)	0.0505 (5)	
H12	0.855831	0.386815	0.863378	0.061*	
C2	0.6266 (2)	0.90036 (14)	0.29204 (16)	0.0546 (5)	
H2B	0.615906	0.975079	0.272312	0.082*	
H2C	0.713615	0.877111	0.291596	0.082*	
H2D	0.617836	0.889302	0.369713	0.082*	
C1	0.3232 (2)	0.41090 (15)	0.02729 (18)	0.0603 (5)	
H1A	0.255752	0.390654	−0.044946	0.090*	
H1B	0.302110	0.380713	0.094094	0.090*	
H1C	0.408703	0.384713	0.025963	0.090*	
C16	1.0104 (2)	0.2356 (2)	0.96441 (19)	0.0743 (7)	
H16A	0.918409	0.214220	0.945995	0.111*	
H16B	1.023357	0.302705	1.006081	0.111*	
H16C	1.066751	0.182152	1.013549	0.111*	

### Atomic displacement parameters (Å^2^)

**Table:**

	*U*^11^	*U*^22^	*U*^33^	*U*^12^	*U*^13^	*U*^23^
O2	0.0477 (7)	0.0351 (6)	0.0389 (6)	0.0019 (5)	−0.0128 (5)	0.0021 (5)
O3	0.0525 (7)	0.0317 (6)	0.0402 (6)	−0.0019 (5)	−0.0114 (5)	0.0026 (5)
O1	0.0521 (7)	0.0372 (7)	0.0488 (7)	−0.0005 (5)	−0.0152 (6)	−0.0056 (5)
O4	0.0602 (8)	0.0525 (8)	0.0453 (7)	0.0195 (6)	0.0174 (6)	0.0221 (6)
N1	0.0403 (7)	0.0353 (7)	0.0342 (7)	0.0050 (6)	0.0017 (6)	0.0049 (6)
C10	0.0353 (8)	0.0315 (8)	0.0324 (8)	−0.0008 (6)	0.0019 (6)	0.0039 (6)
C15	0.0442 (9)	0.0333 (8)	0.0314 (8)	0.0013 (7)	0.0091 (7)	0.0009 (6)
C8	0.0328 (8)	0.0371 (8)	0.0322 (8)	0.0034 (6)	0.0036 (6)	0.0025 (6)
C4	0.0341 (8)	0.0375 (9)	0.0337 (8)	−0.0001 (7)	0.0016 (6)	−0.0034 (6)
C6	0.0354 (8)	0.0323 (8)	0.0308 (8)	−0.0002 (6)	0.0034 (6)	0.0008 (6)
C14	0.0418 (9)	0.0317 (8)	0.0413 (9)	0.0055 (7)	0.0121 (7)	0.0022 (7)
C7	0.0339 (8)	0.0380 (9)	0.0291 (8)	−0.0018 (7)	−0.0011 (6)	−0.0006 (6)
C9	0.0368 (8)	0.0333 (8)	0.0389 (9)	0.0028 (7)	0.0056 (7)	0.0049 (7)
C5	0.0319 (8)	0.0373 (9)	0.0285 (8)	0.0033 (6)	0.0007 (6)	0.0030 (6)
C3	0.0402 (9)	0.0314 (8)	0.0394 (9)	0.0030 (7)	0.0052 (7)	0.0017 (7)
C13	0.0378 (9)	0.0348 (9)	0.0380 (9)	0.0031 (7)	0.0066 (7)	0.0080 (7)
C11	0.0460 (10)	0.0548 (11)	0.0458 (10)	0.0201 (8)	0.0156 (8)	0.0111 (8)
C12	0.0529 (11)	0.0624 (12)	0.0400 (9)	0.0154 (9)	0.0198 (8)	0.0145 (8)
C2	0.0591 (12)	0.0363 (10)	0.0509 (11)	−0.0115 (8)	−0.0089 (9)	0.0027 (8)
C1	0.0658 (13)	0.0427 (11)	0.0568 (11)	−0.0089 (9)	−0.0042 (10)	−0.0113 (9)
C16	0.0816 (16)	0.0896 (17)	0.0569 (13)	0.0299 (13)	0.0291 (11)	0.0395 (12)

### Geometric parameters (Å, º)

**Table:**

O1—C2^i^	3.159 (2)	C6—C7	1.379 (2)
O2—C5	1.3610 (17)	C6—C5	1.394 (2)
O2—H2	0.8200	C14—C13	1.380 (2)
O3—C6	1.3652 (19)	C14—H14	0.9300
O3—C2	1.4193 (19)	C7—H7	0.9300
O1—C4	1.3704 (18)	C9—H9	0.9300
O1—C1	1.415 (2)	C3—H3	0.9300
O4—C13	1.3748 (18)	C13—C12	1.382 (2)
O4—C16	1.420 (2)	C11—C12	1.383 (2)
N1—C9	1.2722 (19)	C11—H11	0.9300
N1—C10	1.4299 (19)	C12—H12	0.9300
C10—C11	1.380 (2)	C2—H2B	0.9600
C10—C15	1.381 (2)	C2—H2C	0.9600
C15—C14	1.381 (2)	C2—H2D	0.9600
C15—H15	0.9300	C1—H1A	0.9600
C8—C7	1.387 (2)	C1—H1B	0.9600
C8—C3	1.398 (2)	C1—H1C	0.9600
C8—C9	1.466 (2)	C16—H16A	0.9600
C4—C3	1.387 (2)	C16—H16B	0.9600
C4—C5	1.388 (2)	C16—H16C	0.9600
			
C5—O2—H2	109.5	C4—C5—C6	119.60 (13)
C6—O3—C2	117.27 (12)	C4—C3—C8	119.96 (15)
C4—O1—C1	117.75 (13)	C4—C3—H3	120.0
C13—O4—C16	117.76 (14)	C8—C3—H3	120.0
C9—N1—C10	116.47 (13)	O4—C13—C14	116.08 (14)
C11—C10—C15	118.47 (14)	O4—C13—C12	124.69 (15)
C11—C10—N1	118.78 (14)	C14—C13—C12	119.22 (14)
C15—C10—N1	122.73 (14)	C10—C11—C12	121.36 (16)
C14—C15—C10	120.54 (15)	C10—C11—H11	119.3
C14—C15—H15	119.7	C12—C11—H11	119.3
C10—C15—H15	119.7	C13—C12—C11	119.69 (16)
C7—C8—C3	120.18 (14)	C13—C12—H12	120.2
C7—C8—C9	122.12 (14)	C11—C12—H12	120.2
C3—C8—C9	117.62 (14)	O3—C2—H2B	109.5
O1—C4—C3	125.06 (15)	O3—C2—H2C	109.5
O1—C4—C5	115.09 (13)	H2B—C2—H2C	109.5
C3—C4—C5	119.85 (14)	O3—C2—H2D	109.5
O3—C6—C7	125.44 (13)	H2B—C2—H2D	109.5
O3—C6—C5	113.65 (13)	H2C—C2—H2D	109.5
C7—C6—C5	120.91 (14)	O1—C1—H1A	109.5
C13—C14—C15	120.67 (15)	O1—C1—H1B	109.5
C13—C14—H14	119.7	H1A—C1—H1B	109.5
C15—C14—H14	119.7	O1—C1—H1C	109.5
C6—C7—C8	119.44 (14)	H1A—C1—H1C	109.5
C6—C7—H7	120.3	H1B—C1—H1C	109.5
C8—C7—H7	120.3	O4—C16—H16A	109.5
N1—C9—C8	124.73 (15)	O4—C16—H16B	109.5
N1—C9—H9	117.6	H16A—C16—H16B	109.5
C8—C9—H9	117.6	O4—C16—H16C	109.5
O2—C5—C4	118.44 (13)	H16A—C16—H16C	109.5
O2—C5—C6	121.96 (14)	H16B—C16—H16C	109.5
			
C9—N1—C10—C11	−120.05 (18)	C3—C4—C5—C6	2.1 (2)
C9—N1—C10—C15	61.7 (2)	O3—C6—C5—O2	−1.2 (2)
C11—C10—C15—C14	0.0 (2)	C7—C6—C5—O2	178.26 (15)
N1—C10—C15—C14	178.20 (15)	O3—C6—C5—C4	177.70 (14)
C1—O1—C4—C3	−4.9 (3)	C7—C6—C5—C4	−2.8 (2)
C1—O1—C4—C5	175.89 (16)	O1—C4—C3—C8	−178.61 (15)
C2—O3—C6—C7	2.4 (2)	C5—C4—C3—C8	0.5 (2)
C2—O3—C6—C5	−178.17 (15)	C7—C8—C3—C4	−2.4 (2)
C10—C15—C14—C13	1.4 (2)	C9—C8—C3—C4	174.26 (15)
O3—C6—C7—C8	−179.66 (15)	C16—O4—C13—C14	−175.25 (18)
C5—C6—C7—C8	0.9 (2)	C16—O4—C13—C12	5.6 (3)
C3—C8—C7—C6	1.7 (2)	C15—C14—C13—O4	179.86 (15)
C9—C8—C7—C6	−174.84 (15)	C15—C14—C13—C12	−1.0 (3)
C10—N1—C9—C8	176.30 (14)	C15—C10—C11—C12	−1.8 (3)
C7—C8—C9—N1	10.4 (3)	N1—C10—C11—C12	179.89 (16)
C3—C8—C9—N1	−166.23 (16)	O4—C13—C12—C11	178.25 (17)
O1—C4—C5—O2	0.2 (2)	C14—C13—C12—C11	−0.8 (3)
C3—C4—C5—O2	−178.98 (14)	C10—C11—C12—C13	2.2 (3)
O1—C4—C5—C6	−178.72 (14)		

Symmetry code: (i) *x*−1/2, −*y*+3/2, *z*−1/2.

### Hydrogen-bond geometry (Å, º)

**Table:**

*D*—H···*A*	*D*—H	H···*A*	*D*···*A*	*D*—H···*A*
O2—H2···N1^i^	0.82	2.21	2.9415 (18)	149
C7—H7···O2^ii^	0.93	2.29	3.2043 (18)	167

Symmetry codes: (i) *x*−1/2, −*y*+3/2, *z*−1/2; (ii) *x*+1/2, −*y*+3/2, *z*+1/2.
